# A computational tool for the design of live attenuated virus vaccine based on microRNA-mediated gene silencing

**DOI:** 10.1186/1471-2164-13-S7-S15

**Published:** 2012-12-07

**Authors:** Duangdao Wichadakul, Wuttichai Mhuantong, Anan Jongkaewwattana, Supawadee Ingsriswang

**Affiliations:** 1 Information Systems Laboratory, National Center for Genetic Engineering and Biotechnology (BIOTEC),113 Thailand Science Park, Phaholyothin Road, Klong 1, Klong Luang, Pathumthani, 12120, Thailand; 2 Enzyme Technology Laboratory, National Center for Genetic Engineering and Biotechnology (BIOTEC),113 Thailand Science Park, Phaholyothin Road, Klong 1, Klong Luang, Pathumthani, 12120, Thailand; 3 Virology and Cell Technology Laboratory, National Center for Genetic Engineering and Biotechnology (BIOTEC),113 Thailand Science Park, Phaholyothin Road, Klong 1, Klong Luang, Pathumthani, 12120, Thailand

## Abstract

**Background:**

The microRNA-based gene-silencing machinery has been recognized as a promising approach to control viral replication and used for improving safety for the live attenuated virus vaccines. The effective host microRNA response elements (MREs) have been incorporated into a virus sequence mainly based on the experimental trials for identifying both microRNA binding sites and effective mutations. The design of MREs for viral genomes or with multiple host microRNAs of interest, then, will be time and cost consuming.

**Results:**

In this paper, we introduced a computational flow that could be used to design MREs of human microRNAs within Influenza A H1N1 virus gene segments. The main steps of the flow includes locating possible binding sites; MREs, of human microRNAs within the viral sequences using a miRNA target prediction tool (miranda), performing various mutations among mismatched binding positions, calculating the binding energy, score, identity, and the effects of changed physical properties of amino acids according to the changed bases in RNA level, and prioritizing the mutated binding sites. The top ranked MREs of human microRNA hsa-miR-93 is consistent with previous literature while other results waited to be experimentally verified. To make the computational flow easily accessible by virologists, we also developed MicroLive, a web server version of the MRE design flow together with the database of miranda-predicted MREs within gene sequences of seven RNA viruses including Influenza A, dengue, hepatitis C, measles, mumps, poliovirus, and rabies. Users may design MREs of specific human microRNAs for their input viral sequences using MRE design tool or optimize the miranda-predicted MREs of seven viruses available on the system. Also, users could design varied number of MREs for multiple human microRNAs to modulate the degree of live vaccine attenuation and reduce the likelihood of escape mutants.

**Conclusions:**

The computational design of MREs helps reduce time and cost for experimental trials. While the flow was demonstrated using human microRNAs and Influenza A H1N1 virus, it could be flexibly applied to other hosts (e.g., animals) and viruses of interest for constructing host-specific live attenuated vaccines. Also, it could be deployed for engineering tissue-specific oncolytic viruses in cancer virotherapeutics. The MicroLive web server is freely accessible at http://www.biotec.or.th/isl/microlive.

## Background

MicroRNAs (miRNAs) are a class of naturally occurring, single-stranded non-coding RNA molecules approximately 21-25 nucleotides in length. It has been shown that miRNAs are, to some extent, complementary to messenger RNA (mRNA) molecules in most, if not all, eukaryotic cells and that their main function is to inhibit gene expression via a number of mechanisms, such as direct mRNA cleavage, translational repression, and deadenylation [1]. In general, miRNAs function by guiding the RNA-induced silencing complex (RISC) to their target sites, resulting in inhibition of mRNA translation followed by deadenylation and rapid degradation of mRNA [2].

The anti-viral role of cellular miRNA was best established by the discovery that an endogenous miRNA, termed miR-32, could suppress the replication of primate foamy virus type-1 (PFV-1) in human cells [3]. During viral replication, miR-32 could bind viral mRNA, with imperfect complementarity, leading to suppression of viral protein synthesis and viral replication. Since then, many more cellular miRNAs have been discovered and its anti-viral effect identified. For instance, a cellular miR-29a in T cells was found to strongly inhibit replication of HIV-1 via a highly conserved target site in the viral genome [4]. Host-encoded miR-101 was found to substantially suppress herpes simplex virus-1 (HSV-1) replication by targeting the 3' UTR of mitochondrial ATP synthase subunit beta [5]. It also has been shown that cellular miR-323, miR-491, and miR-654 could inhibit replication of the H1N1 influenza A virus through binding to the PB1 gene [6]. Cellular miRNAs miR-24 and miR-93 were reported to target viral large protein (L protein) and phosphoprotein (P protein) genes and their decreased expression increased vesicular stomatitis virus (VSV) replication [7]. Taken together, these findings strongly suggest that binding cellular miRNAs to its target sequences in the viral mRNA could be another mechanism by which invading viruses could be counteracted by the host.

Mounting studies have demonstrated that cellular miRNA-mediated anti-viral property is a promising approach for development of attenuated viruses as live vaccines. When the poliovirus was engineered to harbor endogenous miRNA-complementary target sequences, it became highly attenuated as evinced by its poor growth in cells expressing the corresponding miRNA. Moreover, mice infected with these modified viruses exhibited barely detectable sign of disease, and, importantly, they were protected against lethal challenge with a pathogenic poliovirus [8]. Likewise, Perez and colleagues have generated a panel of influenza viruses by incorporating non-avian miRNA response elements (MREs) into the viral nucleoprotein; they could generate H1N1 and H5N1 influenza viruses that were attenuated in mice but not in eggs [9]. Recently, dengue virus replicon was also modified to carry the sequence target of hepatic-specific miR-122 at its 3'-UTR and this virus growth in liver cells was highly restricted [10].

Besides improving safety for live attenuated virus vaccines, the engineering of viruses with the miRNA response elements produces potent oncolytic viruses which are tissue-specifically safe. Cawood and colleges engineered an adenovirus 5 (Ad5) with the inserted binding sites of hepatocyte-specific miR-122 within the 3'-UTR of E1A transcription cassette which resulted in liver-safe oncolytic virus with full activity in cancer cells [11]. Herpes Simplex Virus-1 (HSV-1) was engineered to incorporate the binding sites of miR-143 and miR-145 into the 3'-UTR of ICP4 gene. This significantly reduced virulence to normal tissues but maintained the killing of prostate cancer cells [12]. Edges and colleagues incorporated the complementary sequence of let-7 microRNA within Vesicular Stomatitis Virus (VSV), a rhabdovirus, to eliminate undesirable replication and toxicity in normal cells but still grow in cancer cells [13]. Kelly and colleagues inserted miR-125 target sequences into the 3'-UTR of the polymerase (L) gene of the Vesicular Stomatitis Virus (VSV) to reduce the neurotoxicity [14]. Coxsackievirus A21 (CVA21), a picornavirus, was also engineered with target elements of the muscle-specific miR-1, miR-133a, and miR-206 to retain the oncolytic activity but protect myositis [15].

These studies clearly indicate that, with the advent of reverse genetics technology, it is possible to manipulate the infectious clones of virus of interest by incorporating the miRNA target sequences of choice into the viral genome and construct recombinant viruses for the development of live attenuated vaccines or tissue-specific oncolytic viruses for cancer virotherapy.

The inserted positions of MREs into a viral genome could be varied from 3'-UTR to open reading frames (ORFs) with different complexities (e.g., the highly-structured RNA, the possibly available RNA pseudoknots, the cyclization/conserved sequences and stem-loop regions in 3'-UTR, or the effects of physical properties according to mutated bases in RNA level of the ORFs). Without computer-aided tools, the proper MRE positions and mutations would be mainly identified by experimental trials. To ease the design of MREs and to reduce time for wet-lab experiments, here, we proposed a computational flow and a web server for designing MREs of human miRNAs that could be incorporated into the open reading frames (ORFs) of a viral genome with efficacy, and having no or least effects on physical properties of amino acids due to mutated bases in RNA level. The results of the entire computational flow was demonstrated via the designed MREs within gene segments of Influenza A H1N1 virus for a set of human miRNAs. The developed web server consists of two parts: 1) the MRE design tool that implemented the computational flow with web interfaces allowing users to customize binding parameters and mutate binding positions with score recalculations, and 2) the database of seven RNA viruses with miranda-predicted MREs that could be further optimized and prioritized as effective MREs for wet-lab experiments.

## Materials and methods

### Data compilation

To demonstrate the entire flow of MRE design, sequences of previously reported human (*Homo sapiens*) miRNAs were extracted from miRBase [16,17] release 14. The total 122 human miRNAs of interest remained after verifying their conservation with mouse (*Mus musculus*) but not chicken (*Gallus domesticus*) as Influenza virus was normally cultivated in eggs. The number of remaining miRNAs was further reduced according to their ubiquitous expression in various tissues. The total 19 out of 122 miRNAs expressed in more than five tissues were extracted from supplemental data of [18] (see Additional file 1 for the list of these miRNAs) and selected as host miRNAs of interest for MRE design. The Influenza RNA sequences (influenza.cds.gz) was downloaded from ftp://ftp.ncbi.nih.gov/genomes/INFLUENZA/ on January, 2010. Sequences of Influenza A H1N1 virus were grouped into eight files with 1019, 1019, 1015, 1051, 1022, 1047, 1035, and 1017 sequences according to gene segments PB2, PB1, PA, HA, NP, NA, M, NS.

To build a database of miranda-predicted MREs, Influenza A sequences of H1N1, H1N2, H2N2, and H3N2 viruses were downloaded from Influenza Virus Resource at NCBI (http://www.ncbi.nlm.nih.gov/genomes/FLU/FLU.html) at the end of August 2010. Other virus sequences including dengue virus (types 1-4), hepatitis C virus (genotypes 1-6), measles, mumps, human poliovirus (1-3), and rabies were collected from NCBI viral genomes resource (http://www.ncbi.nlm.nih.gov/genomes/GenomesHome.cgi?taxid=10239) during August 31-September 3, 2010 (see Additional file 2 for the number of compiled viral sequences). The compiled sequences of the same gene or gene segment of a specific virus (e.g., gene segment 5 of Influenza A H1N1 or NS2A gene of dengue virus type 1), were grouped together, cleaned and then aligned using MUSCLE [19]. These alignment results were prepared as a part of the online database for helping users choose the predicted MREs for further optimization according to their conserved regions over sequence population. All human miRNAs were extracted from miRBase release 16.

### Computational identification and prioritization of host MREs

**Figure 1:**
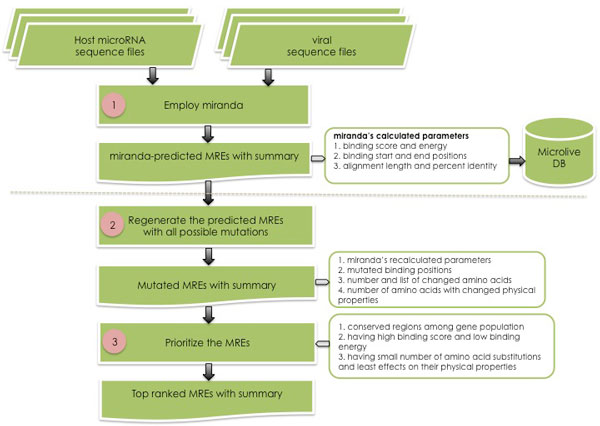
**Overall workflow for the computational identification of potential miRNA response elements (MREs)**.

The computational flow used to design potential miRNA response elements (MREs) consists of three main phases as demonstrated in Figure 1. First phase, the possible binding sites (MREs) of host miRNAs on viral sequences were predicted by miranda [20,21] (version v3.3a), an miRNA target prediction tool, with default parameter setting and the binding energy cutoff was less than or equal to -15 KCal/Mol. Virologists might simply incorporate these MREs into their input viral sequences. This however does not guarantee the binding effectiveness. Second phase, the miranda-predicted MREs were regenerated with all combinations of mutations from the non-complementary to complementary positions with the recalculation of binding score and energy, the measurement of amino acid substitutions and the effects on their physical properties (e.g., hydrophobicity, polarity and/or charge) due to the mutated bases in the RNA level. Third phase, the mutated MREs would be prioritized; they will be top ranked if 1) located on conserved open reading frames (ORFs) of the same gene across sequence population, 2) having high binding score and low binding energy against the host miRNA, and 3) having small number of amino acid substitutions and getting no or least effects on their physical properties. Multiple MREs for the same miRNAs and/or different miRNAs will be also summarized as alternative MREs that could be deployed simultaneously to alleviate the effects of viral mutation across the generations. All these steps were implemented as a set of Python codes.

To make the MRE design tool publicly accessible and usable by virologists, we also implemented the computational flow as a web server called MicroLive and a database of miranda-predicted MREs, including binding sites, binding score, binding energy, and percent identity, resulted from first phase of the computational flow, of all human miRNAs from miRBase release 16 within seven RNA viruses. This database could be the starting point of virologists to try the online MRE design tool to optimize and prioritize the MREs.

## Results and discussions

### Prioritized MREs for influenza A H1N1 virus

This section demonstrates the final result generated by running the entire steps of the computational flow to find the potential MREs for a set of human microRNAs against eight segments of Influenza A H1N1 2009 virus (see Materials and Methods section). Nine distinct binding sites of six human miRNAs, hsa-miR-25, hsa-miR-92a, hsa-miR-93, hsa-miR-99b, hsa-miR-191, and hsa-miR-503 were top ranked by the prioritization with binding energy lower than -37 KCal/Mol and having at most two amino acid substitutions without effects on physical properties (see Additional file 3). These MREs were located within gene segments 2, 4, 5, 6, or 7 of Influenza A H1N1 virus. Among them, an MRE of hsa-miR-93 with binding position 772-794 nt (from the start codon) was identified within segment 5 (NP) of the Influenza A H1N1 virus and consistent with *Site 2 *in [9]. *Site *1 in [9] was not identified by our binding energy cutoff. Human miRNAs hsa-miR-99b and hsa-miR-503 has an MRE with only single amino acid substitution on segment 6 (NA) and segment 4 (HA), respectively. In addition, hsa-miR-25 has multiple MREs on gene segment 2 (PB1). These prioritized MREs are considered potential MREs for wet-lab experiments.

### MRE design tool

**Figure 2:**
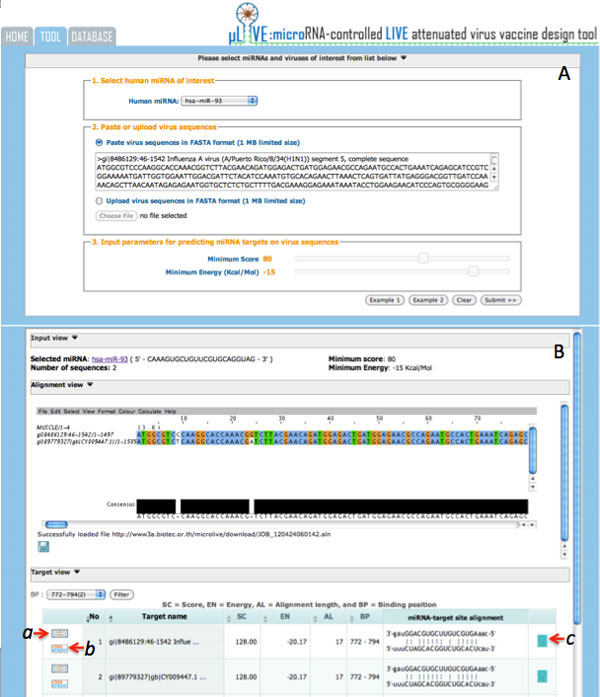
**The first phase of MRE design tool interface: (A) parameter setting of Example 1, and (B) results of input sequence alignment and potential binding sites of the selected human miRNA**.

**Figure 3:**
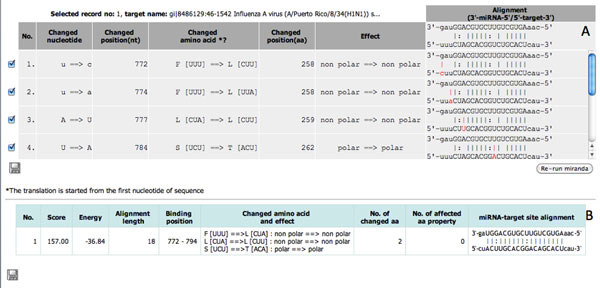
**The second phase of MRE design tool interface: (A) interface for mutating mismatched positions, (B) the recalculated binding score, energy, and the effects of amino acid substitutions according to mutated positions**.

**Figure 4:**
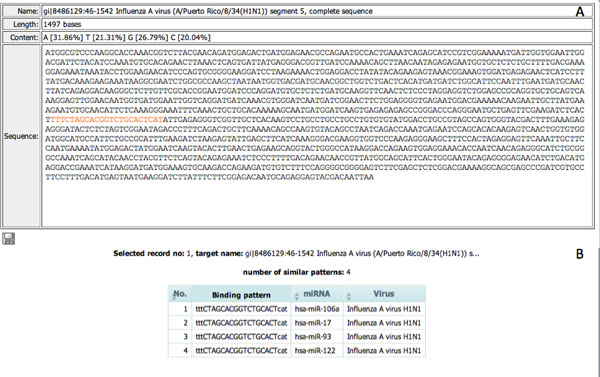
**The additional information of an input sequence containing miranda-predicted MREs: (A) sequence detail with highlighted target sites and percent of nucleotide content, (B) list of other miRNAs targeting the same site**.

The MRE design tool implemented the computational flow of MRE design into a web-based application of two main steps: I) identify the possible binding sites of a human miRNA within input virus sequences, II) design MREs from the binding sites of interest. In the first step, users need to 1) select a specific human miRNA of interest, 2) paste or upload a set of viral sequences of the same gene or gene segment with the maximum file size limited to 1MB, and 3) specify the cutoff parameters for keeping the possible miRNA-virus binding sites. From *Example 1 *(see Figure 2A), with selected human miRNA hsa-miR-93 and two input sequences of segment 5 (NP), gi|8486129:A/Puerto Rico/8/34(H1N1) and gi|89779327: A/Puerto Rico/8/34(H1N1) of Influenza A virus with minimum score and maximum energy as 80 and -15 KCal/Mol, we got 18 records of input sequences with nine binding sites (see Figure 2B). From here, we could filter the list of records according to a specific conserved binding site and start the MRE design step. We selected the binding site 772-794 in this example as it has the highest score and lowest binding energy and is conserved between the two input sequences. The conserved sites could be explored through the "Alignment View" visualized using Jalview [22]. Under the "Target View", the click on "Design MREs" button *a *of any sequences having the conserved binding site will open a "Show mutation" window (Figure 3). Here, users could iteratively mutate mismatched binding positions, rerun miranda, and explore the recalculated binding score, binding energy, the number of changed amino acids, and the effects on their physical properties. Users may export the mutated sequence if required. The click on "View sequence and detail" button *b *(Figure 2B) will open a new window (Figure 4A) displaying the selected sequence with highlighted target sites and percent of nucleotide content whereas the click on "See other miRNAs that target the same binding site" button *c *will open a new window (Figure 4B) with a list of miRNAs with the same binding site. Users could use some of these miRNAs as alternatives for the rerun of MRE design.

### Database of miranda-predicted human MREs

#### Search utility

The MicroLive database contains the list of miranda-predicted MREs of all human miRNAs in miRBase release 16 within seven RNA viruses: Influenza A (H1N1, H1N2, H2N2, and H3N2), dengue virus (types 1-4), hepatitis C virus (genotypes 1-6), measles, mumps, poliovirus (1-3), and rabies. To search these MREs within a virus, users need to select a gene or gene segment of a virus and a specific human miRNA of interest, customize the binding parameters including the minimum binding score, maximum binding energy, and alignment length that represent the binding affinity of the chosen miRNA on the MREs, and submit the search request. The selected segment 5 (NP) of Influenza A H1N1 virus in Bangkok with selected human miRNA hsa-miR-93 and default binding parameters setting (e.g., minimum score, maximum energy, and alignment length is equal to 80, -15 KCal/Mol, and 10 bp, respectively), resulted in five distinct NP genes, each with two binding positions. From here, users could select one of the binding positions of interest based on their conservation in the "Alignment View," and click on "Design MREs" button on the left of any sequences in the group for trying the MRE design step as described in the design tool section.

#### Conserved positions of miranda-predicted MREs

This section gives examples of the potential start sites of miranda-predicted MREs within Influenza A viruses, dengue types 1-4, and rabies according to their conserved start binding positions of specific miRNAs within or across gene sets.

##### Influenza A viruses

The numbers of human miRNAs identified with the miranda-predicted MREs on specific viral genes or gene segments were varied (see Additional file 4). We found two human miRNAs hsa-miR-637 and hsa-miR-760 having MREs (with binding energy <= -30 KCal/Mol) within gene segment 5 (NP) across the four Influenza A H1N1, H1N2, H2N2, and H3N2 viruses. The start binding position 521 of hsa-miR-637 within the segment was highly conserved among gene populations of H1N2, H2N2, and H3N2 viruses. Additionally, the start binding positions 537 and 560 of hsa-miR-760 of the three viruses were also highly conserved. All these start binding positions were specific to the corresponding human miRNAs. All viruses except H1N1 also shared MREs of another human miRNA hsa-miR-657 with highly conserved binding position 639. Specific to Influenza A H1N1 virus, the start binding positions 1161, 65, and 1190 of hsa-miR-1538, hsa-miR-4254, and hsa-miR-4286 were highly conserved among more than thousand sequences of segment 5 within the population. These positions were considered potential start positions for the design of MREs.

##### Dengue viruses

With the same binding energy cutoff, we found two human miRNAs hsa-miR-370 and hsa-miR-661 having MREs within NS1 genes of all types of dengue virus. The start binding position 771 of hsa-miR-370 was highly conserved among gene population of types 1-3 while hsa-miR-661 had a highly conserved start binding position 789 among gene population of all types. Both start binding positions were specific to the corresponding miRNAs. Besides the conserved start position 771, positions 139 of hsa-miR-1470, 141 of hsa-miR-3622a-3p, 148 of hsa-miR-485-3p, and 582 of hsa-miR-770-5p were highly conserved among more than one thousand sequences of NS1 genes in type 1 while position 137 was predicted start binding position of both hsa-miR-1236 and hsa-miR-1249. The start binding positions 304 of hsa-miR-762 and 308 of hsa-miR-3677 were highly conserved among more than five hundred NS1 genes in type 2 while positions 765 of hsa-miR-637 and 773 of hsa-miR-3663-5p were highly conserved among almost three hundred NS1 sequences in dengue type 3.

##### Rabies

More than two hundred nucleoprotein (NP) genes of rabies virus were predicted with highly conserved start binding positions 122 of hsa-miR-939, 359 of hsa-miR-770-5p, and 820 of hsa-miR-2277-3p and hsa-miR-638. Interestingly, more than one hundred of phosphoprotein (P) genes contained two conserved positions 530 and 531 which were targeted by multiple miRNAs. Start position 530 was targeted by hsa-miR-3131, hsa-miR-4298, and hsa-miR-939 while position 531 was targeted by hsa-miR-134 and hsa-miR-3180-3p. Likewise, six start binding positions found conserved across hundred of glycoprotein genes. Top three of them were positions 87 of hsa-miR-3151 and hsa-miR-4259, 1331 of hsa-miR-611, and 1478 of hsa-miR-1236.

## Conclusions

This paper presents a computational flow that helps virologists design the potential miRNA response elements (MREs) for constructing safer live attenuated virus vaccines. The designed MREs of selected human miRNAs within Influenza A H1N1 virus demonstrated the usefulness of the proposed computation to reduce the experimental time and cost with the consistent result comparing with previous literature. The MRE design tool on the MicroLive web server enables users to interactively find and optimize the potential MREs for a specific human miRNA within users' input viral sequences of interest. The provided database allows users to explore the miranda-predicted MREs for several RNA viruses and try the MRE design tool based on the system-provided sequences. While the demonstration and implementation were based on human miRNAs and Influenza A H1N1 virus, the proposed computation could be flexibly applied to design MREs for engineering tissue-specific oncolytic viruses or for constructing live attenuated virus vaccines for other hosts of interest such as animals.

## Availability and requirements

• MicroLive is freely available at http://www.biotec.or.th/isl/microlive/.

• The multiple sequence alignment of the same gene requires Jalview java applet to display.

## Competing interests

The authors declare that they have no competing interests.

## Authors' contributions

DW designed the computational flow for MRE identification, performed the MRE design and analysis for Influenza A H1N1 virus, and drafted the manuscript. WM designed and implemented the MicroLive web server and database. AJ and SW helped improve the criteria for MRE ranking, MRE design flow, and the final manuscript. All co-authors read and approved the final manuscript.

## Supplementary Material

Additional file 1 **List of ubiquitously expressed human miRNAs that are conserved with mouse but not chicken**. Click here for file

Additional file 2 **Number of compiled viral sequences in MicroLive database**. Click here for file

Additional file 3 **List of human MREs identified within gene segments of Influenza A H1N1 virus with binding energy lower than -37 KCal/Mol and having at most two amino acid substitutions without effects on physical properties**. Click here for file

Additional file 4 **Number of human miRNAs identified with the miranda-predicted MREs within specific viral genes or gene segments**. Click here for file

## References

[B1] Cullen BR Viruses and microRNAs: RISCy interactions with serious consequences Genes Dev 2011 25 18 1881 1894 10.1101/gad.17352611 21896651 PMC3185961

[B2] Huntzinger E Izaurralde E Gene silencing by microRNAs: contributions of translational repression and mRNA decay Nat Rev Genet 2011 12 2 99 110 10.1038/nrg2936 21245828

[B3] Lecellier CH Dunoyer P Arar K Lehmann-Che J Eyquem S Himber C Saib A Voinnet O A cellular microRNA mediates antiviral defense in human cells Science 2005 308 5721 557 560 10.1126/science.1108784 15845854

[B4] Nathans R Chu CY Serquina AK Lu CC Cao H Rana TM Cellular microRNA and P bodies modulate host-HIV-1 interactions Mol Cell 2009 34 6 696 709 10.1016/j.molcel.2009.06.003 19560422 PMC2763548

[B5] Zheng SQ Li YX Zhang Y Li X Tang H MiR-101 regulates HSV-1 replication by targeting ATP5B Antiviral Res 2011 89 3 219 226 10.1016/j.antiviral.2011.01.008 21291913

[B6] Song L Liu H Gao S Jiang W Huang W Cellular microRNAs inhibit replication of the H1N1 influenza A virus in infected cells J Virol 2010 84 17 8849 8860 10.1128/JVI.00456-10 20554777 PMC2919005

[B7] Otsuka M Jing Q Georgel P New L Chen J Mols J Kang YJ Jiang Z Du X Cook R Hypersusceptibility to vesicular stomatitis virus infection in Dicer1-deficient mice is due to impaired miR24 and miR93 expression Immunity 2007 27 1 123 134 10.1016/j.immuni.2007.05.014 17613256

[B8] Barnes D Kunitomi M Vignuzzi M Saksela K Andino R Harnessing endogenous miRNAs to control virus tissue tropism as a strategy for developing attenuated virus vaccines Cell Host Microbe 2008 4 3 239 248 10.1016/j.chom.2008.08.003 18779050 PMC2605097

[B9] Perez JT Pham AM Lorini MH Chua MA Steel J tenOever BR MicroRNA-mediated species-specific attenuation of influenza A virus Nat Biotechnol 2009 27 6 572 576 10.1038/nbt.1542 19483680

[B10] Lee TC Lin YL Liao JT Su CM Lin CC Lin WP Liao CL Utilizing liver-specific microRNA-122 to modulate replication of dengue virus replicon Biochem Biophys Res Commun 2010 396 3 596 601 10.1016/j.bbrc.2010.04.080 20412785

[B11] Cawood R Chen HH Carroll F Bazan-Peregrino M van Rooijen N Seymour LW Use of tissue-specific microRNA to control pathology of wild-type adenovirus without attenuation of its ability to kill cancer cells PLoS Pathog 2009 5 5 e1000440 10.1371/journal.ppat.1000440 19461878 PMC2678247

[B12] Lee CYF Rennie PS Jia WWG MicroRNA regulation of oncolytic herpes simplex virus-1 for selective killing of prostate cancer cells Clin Cancer Res 2009 15 16 5126 5135 10.1158/1078-0432.CCR-09-0051 19671871

[B13] Edge RE Falls TJ Brown CW Lichty BD Atkins H Bell JC A let-7 MicroRNA-sensitive vesicular stomatitis virus demonstrates tumor-specific replication Mol Ther 2008 16 8 1437 1443 10.1038/mt.2008.130 18560417

[B14] Kelly E Nace R Barber G Russell S Attenuation of VSV-encephalitis through microRNA-targeting J Virol 2009 10.1128/JVI.01788-09 PMC2812322 19906911

[B15] Kelly E Hadac E Greiner S Russell S Engineering microRNA responsiveness to decrease virus pathogenicity Nature Medicine 2008 10.1038/nm.1776 18953352

[B16] Griffiths-Jones S Grocock RJ van Dongen S Bateman A Enright AJ miRBase: microRNA sequences, targets and gene nomenclature Nucleic acids research 2006 34 Database D140 144 16381832 10.1093/nar/gkj112 PMC1347474

[B17] Griffiths-Jones S Saini HK van Dongen S Enright AJ miRBase: tools for microRNA genomics Nucleic acids research 2008 36 Database D154 158 17991681 10.1093/nar/gkm952 PMC2238936

[B18] Landgraf P Rusu M Sheridan R Sewer A Iovino N Aravin A Pfeffer S Rice A Kamphorst AO Landthaler M A mammalian microRNA expression atlas based on small RNA library sequencing Cell 2007 129 7 1401 1414 10.1016/j.cell.2007.04.040 17604727 PMC2681231

[B19] Edgar RC MUSCLE: multiple sequence alignment with high accuracy and high throughput Nucleic acids research 2004 32 5 1792 1797 10.1093/nar/gkh340 15034147 PMC390337

[B20] Enright AJ John B Gaul U Tuschl T Sander C Marks DS MicroRNA targets in Drosophila Genome Biol 2003 5 1 R1 10.1186/gb-2003-5-1-r1 14709173 PMC395733

[B21] John B Enright AJ Aravin A Tuschl T Sander C Marks DS Human MicroRNA Targets PLoS Biol 2004 2 11 e363 10.1371/journal.pbio.0020363 15502875 PMC521178

[B22] Waterhouse AM Procter JB Martin DMA Clamp M Barton GJ Jalview Version 2--a multiple sequence alignment editor and analysis workbench Bioinformatics 2009 25 9 1189 1191 10.1093/bioinformatics/btp033 19151095 PMC2672624

